# The effect of chemotherapeutic drugs on human B lymphocytes

**DOI:** 10.1186/2051-1426-1-S1-P252

**Published:** 2013-11-07

**Authors:** Ali Zirakzadeh, Jin Hu, Johan Kinn, Per Marits, Amir Sherif, Ola Winqvist

**Affiliations:** 1 Karolinska Institutet, Department of Medicine, Unit of Translational Immunology L2:04, Stockholm, Sweden; 2 Umeå University Hospital, Department of Surgical and Perioperative Sciences, Urology and Andrology, Umeå, Sweden

## Introduction

Cancer is currently treated by a combination of therapies, including chemotherapy. As a side effect, chemotherapy is believed to suppress the host immune system. Combination of immunotherapy and chemotherapy has been reported to be correlated with improvement of survival. However this combination needs careful planning in order to achieve a synergistic effect. In this study we have performed a flow cytometry analysis of lymphoblast transformation to investigate the effects of three conventional chemotherapeutic drugs, cisplatin, doxorubicin and irinotecan on human B cells and their role as APC.

## Methods

CD19+ B and CD4+ T lymphocytes were isolated from PBMC of healthy donors, using MACS separation beads. They were then incubated separately overnight, either alone or with one of the chemotherapeutic drugs. The next day, the treated CD19+ and CD4+ cells (at 1: 2 ratio) were mixed and treated with Staphylococcal enterotoxin B. The samples were incubated for a 4-day period for blast transformation. Flow cytometry was used to study the blasts in assay of specific cell mediated immune response.

## Result

We investigated antigen presenting ability of human B cells under influence of cisplatin, doxorubicin and irinotecan chemo drugs. We used CD4+ lymphocytes as responder cells. When B cells treated with doxorubicin, was mixed with untreated CD4+ T cells, an increase of CD4+ lymphoblast was observed at day 4. Furthermore, when CD19+ cells were treated with Doxorubicin overnight, the percentage of CD19+CD86+ cells were increased compared to control which suggested an amplified signal of antigen presentation. B cells were then treated with doxorubicin, and incubated with or without anti-CD86 blocking antibody. They were then co-cultured with CD4+ T cells in presence of Staphylococcal enterotoxin B. After four days the lymphoblasts were studied with flow cytometry. In the sample with blocked CD86 molecules, CD4+ exhibited a slight reduction in lymphoblast formation. This indicates that CD86 contributes, at least partly, to the increased T cell activation by doxorubicin-treated B cells.

## Discussion

We conclude that the doxorubicin enhance antigen-presenting ability of human B cells. Developing time and dose schedules may increase the effectiveness of combining chemotherapy and immunotherapy.

